# Insights from bidirectional Mendelian randomization: Evaluating the influence of circulating inflammatory cytokines on prostatitis

**DOI:** 10.1097/MD.0000000000042438

**Published:** 2025-05-09

**Authors:** Jiaqi Ma, Lilei Xu, Chuanlong Zhou, Zhe Shen, Kean Zhu, Xianming Lin

**Affiliations:** a Third Clinical Medical College, Zhejiang Chinese Medical University, Hangzhou, China; b Department of Acupuncture, Third Affiliated Hospital of Zhejiang Chinese Medical University, Hangzhou, China.

**Keywords:** acupuncture, biomarkers, causal relationship, inflammation, Mendelian randomization, prostatitis

## Abstract

Research on prostatitis has primarily focused on inflammatory cytokines in semen or prostatic secretions, with relatively few studies investigating circulating inflammatory cytokines. To explore the relationship between prostatitis and circulating inflammatory cytokines, this study employed bidirectional two-sample Mendelian randomization (MR) to assess the potential associations between prostatitis and 91 circulating inflammatory cytokines. We performed bidirectional MR to explore causal links between 91 circulating inflammatory cytokines and prostatitis. Data were sourced from 14,824 individuals of European ancestry and the Finngen database for prostatitis. The inverse variance-weighted (IVW) method was the primary tool, complemented by MR-Egger, weighted median, weighted mode, and MR-PRESSO to enhance result robustness. Heterogeneity and pleiotropy evaluations were conducted, and GO/KEGG enrichment analyses were used to explore the biological pathways linked to these inflammatory factors and prostatitis. The MR results revealed that Interleukin-10 receptor A (IL-10RA), Natural Killer Cell Receptor 2B4 (CD244), and urokinase-type plasminogen activator (uPA) were identified as risk factors for prostatitis (IVW_IL-10RA_: OR = 1.242, 95% CI: 1.043–1.478, *P *= .015; IVW_CD244_: OR = 1.143, 95% CI: 1.002–1.305, *P *= .047; IVW_uPA_: OR = 1.141, 95% CI: 1.009–1.290, *P *= .035). Conversely, Interleukin-12B (IL-12B) exhibited a protective effect against prostatitis (IVW_IL-12B_: OR = 0.909, 95% CI: 0.842–0.981, *P *= .014). Moreover, reverse MR analysis results indicate that prostatitis decreases plasma levels of chemokine (C-C motif) ligand 23 (CCL23), IL-5, and TNF-related activation-induced cytokine (TRANCE) (IVW_CCL23_: OR = 0.949, 95% CI: 0.906–0.993, *P *= .025; IVW_IL-5_: OR = 0.938, 95% CI: 0.890–0.988, *P *= .016; IVW_TRANCE_: OR = 0.947, 95% CI: 0.905–0.992, *P *= .021). This bidirectional MR study identified potential causal links between 7 circulating inflammatory cytokines and prostatitis, offering insights into its pathogenesis and potential targets for future therapies.

## 1. Introduction

The prevalence of inflammatory diseases of the prostate is high,^[1]^ with chronic prostatitis/chronic pelvic pain syndrome (CP/CPPS) being the most common, accounting for approximately 90% of all cases of prostatitis.^[2]^ It ranks third in prevalence among prostate diseases, following benign prostatic hyperplasia and prostate cancer.^[3,4]^ Currently, the etiology of CP/CPPS remains unclear, but hidden pathogen infections, abnormal autoimmune responses, neural stimulation, and psychological factors are believed to be associated with its onset.^[4]^ Patients often experience pain, lower urinary tract symptoms, sexual dysfunction, and psychological disorders.^[3]^ Moreover, studies have shown a significant association between CP/CPPS and male infertility.^[5]^ Patients diagnosed with CP/CPPS frequently demonstrate reduced semen quality, which may be closely related to inflammatory processes and pathogen infections.^[6,7]^ Elevated levels of inflammatory cytokines, such as interleukins and tumor necrosis factor, are often detected in the semen and blood of male infertility patients,^[8]^ which may impair spermatogenesis and affect sperm function. To elucidate the reasons for the persistent chronic inflammation in CP/CPPS, previous research has focused on investigating inflammatory cytokines in the semen or in prostatic secretions of patients, neglecting the situation of inflammatory cytokines in the blood plasma.^[9–11]^ Therefore, the reference value of these research results is limited for evaluating systemic psychological and neurological symptoms in CP/CPPS.

Mendelian randomization (MR) analysis is an analytical method similar to a randomized controlled trial, which uses instrumental variables (IVs) from non-experimental datasets to assess causality. MR relies on the random allocation of alleles during genetic inheritance to avoid potential confounding factors and reverse causality, thus providing more reliable causal analysis results.^[12]^ This study utilized genetic variants from genome-wide association studies (GWAS) of 91 inflammatory cytokines, conducting bidirectional two-sample MR analyses to explore their causal relationships with prostatitis.

## 2. Materials and methods

### 2.1. MR assumptions

**Figure 1.**
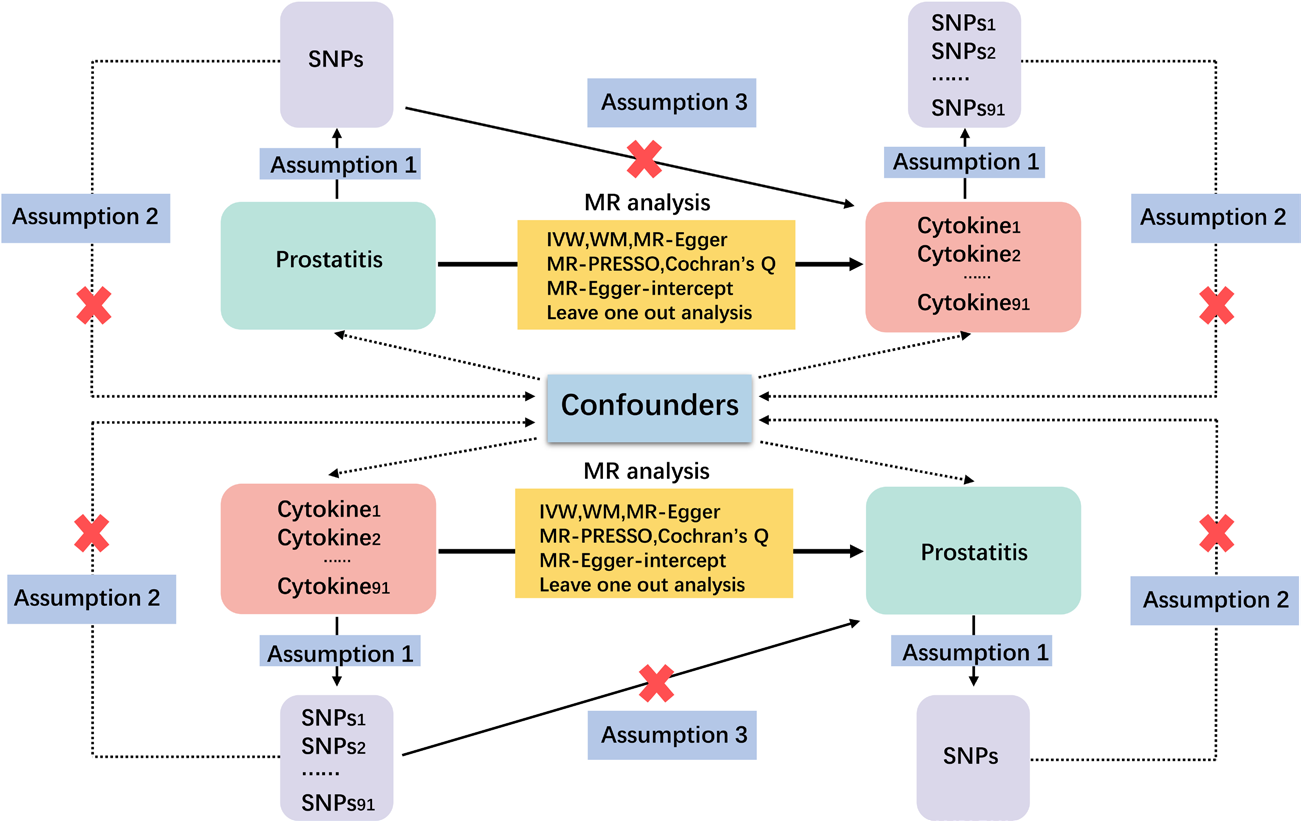
Overview of research design. Assumption1: IVs must be associated with the risk factor. Assumption2: IVs must be independent of any confounding factors between the risk factor and the outcome. Assumption3: IVs must only affect the outcome through the risk factor instead of any other pathway. IV = instrumental variables, MR analysis = Mendelian Randomization analysis, SNPs = single nucleotide polymorphisms.

We utilized 91 GWAS to filter single nucleotide polymorphisms (SNPs) associated with 91 inflammatory cytokines and prostatitis. The filtered SNPs were then utilized as IVs in MR analysis. The selected IVs must meet 3 fundamental assumptions: IVs must be associated with the risk factor; IVs must be independent of any confounding factors between the risk factor and the outcome; and IVs must only affect the outcome through the risk factor instead of any other pathway.^[13]^ Any IVs violating these 3 fundamental assumptions were excluded. This study proceeded with the following steps to complete the MR analysis. Initially, 91 inflammatory cytokines were used as exposures to investigate their potential causal relationship with prostatitis risk. Subsequently, prostatitis was set as the exposure to explore the potential reverse causal relationship with inflammatory cytokines. An overview of the research design is provided in Figure 1.

### 2.2. Instrumental variable selection

Firstly, we set the SNP threshold associated with inflammatory cytokine phenotypes at *P* < 5 × 10^−6^.^[14]^ Secondly, we performed clump processing (kb = 10000, *r*^2^ = 0.001)^[14]^ on these selected SNPs to alleviate the impact of linkage disequilibrium. For SNPs associated with prostatitis, we applied the same threshold at *P* < 5 × 10^−6^ and conducted identical clump processing (kb = 10000, *r*^2^ = 0.001). F-statistic computation was conducted for each SNP after removing linkage disequilibrium. SNPs with an F-statistic lower than 10 were removed to reduce bias from weak instrumental variables. Moreover, we searched for the secondary phenotype of the remaining SNPs on the Phenoscanner database (https://www.phenoscanner.medschl.cam.ac.uk) and excluded SNPs potentially associated with confounding factors.

### 2.3. Data source

The GWAS of 91 inflammatory cytokines originated from a study by Zhao Jinghua et al published in 2023,^[15]^ which conducted a genome-wide protein quantitative trait locus mapping analysis on 14,824 European ancestry participants. The study utilized protein quantitative trait locus information within ± 1 Mb of 91 plasma inflammatory cytokine genes for 91 GWAS. The summary statistics data of GWAS for prostatitis were obtained from Finngen (https://r10.finngen.fi/), which included 4160 cases and 130,139 controls, totaling 21,278,463 SNPs. Prostatitis patients were identified based on International Classification of Diseases, Tenth Revision codes. The selected GWAS were conducted on European ancestry participants, thus mitigating bias due to population stratification.

### 2.4. Statistical analysis

The study applied R (version 4.3.2) environment and conducted Mendelian Randomization analysis using the TwoSampleMR (version 0.5.8) and MRPRESSO (version 1.0) packages. In the MR analysis, to investigate the relationship between inflammatory cytokines and prostatitis, we employed inverse variance-weighted (IVW), MR-Egger, weighted median, weighted mode, and MR-Presso methods for analysis. In the reverse MR analysis, the same methods were used to explore the causal relationship between prostatitis and inflammatory cytokines. IVW is a method in MR with strong capability of assessing causal relationships.^[16]^ It requires all SNPs to meet the basic assumptions of instrumental variables and estimates their effects.^[17]^ Due to its robust testing capability, the results of the IVW method were considered as the primary results of this study. However, the assumption that all genetic variations are effective instrumental variables may be challenging in practice. Therefore, MR-Egger, weighted median, weighted mode, and MR-Presso methods were used to assist in assessing IVW estimates. Furthermore, the results of IVW require SNPs to exhibit the same level of horizontal pleiotropy to avoid the influence of confounding factors. Because of the limitations of the IVW method, it is necessary to test for heterogeneity and pleiotropy in sensitivity analyses. Heterogeneity was assessed by evaluating Cochrane’s Q statistic in the MR-IVW and MR-Egger models (*P* > .05 indicates no heterogeneity). For pleiotropy, the MR-Egger method was used, with the criterion being the absence of a significant difference from zero in the intercept term (*P* < .05), indicating no pleiotropy. Additionally, MR-PRESSO was utilized to detect and remove outlier SNPs, correct for horizontal pleiotropy, and provide more reliable causal estimates. Lastly, leave-one-out analysis was conducted by sequentially removing each SNP to explore the impact of individual SNPs on causal associations.

### 2.5. Gene ontology (GO) and kyoto encyclopedia of genes and genomes (KEGG) pathway analyses

The results of the MR analysis were selected for enrichment analysis to interpret the functions of related genes from the perspectives of biological processes, cellular components (CC), and molecular functions. GO and KEGG analyses were used to investigate the biological pathways associated with prostatitis induction by inflammation factor genes that were positive in the MR analysis.

### 2.6. Ethics statement

Since all these data are publicly available, ethical approval is not required for this study.

## 3. Result

### 3.1. The selection results of instrumental variables

In the MR analysis, we selected SNPs from the dataset of 91 inflammatory cytokines according to the above 3 basic assumptions, and here only positive results are presented. Taking IL-10RA cytokine as an example, 11 SNPs were selected from the corresponding GWAS dataset, which are strongly associated with IL-10 cytokine (*P* < 5 × 10^−6^). Following that, the linkage disequilibrium of the SNPs (kb = 10,000, *r*^2^ = 0.001) was removed, and they were intersected with the prostatitis GWAS dataset. After harmonizing 11 SNPs, a palindromic SNP (rs1406714) was removed. Using the PhenoScanner database, SNPs associated with other risk factors for prostatitis such as “smoking, drinking, BMI,”^[14]^ or with the second phenotype associated with other prostate diseases were removed. Because (rs10091841) was associated with BMI, it was removed. The F values of the remaining SNPs were examined, ranging from 20.45 to 47.99, indicating no weak instrumental variables. MR-PRESSO analysis did not find any outliers. Finally, we obtained 9 SNPs to serve as instrumental variables for evaluating the causal relationship between the IL-10RA cytokine and prostatitis. For detailed information on SNPs related to other positive results, please refer to Table 1. In the reverse MR analysis, the prostatitis GWAS was processed according to the same parameters (*P* < 5 × 10^−6^, kb = 10000, *r*^2^ = 0.001). The SNPs removed through the PhenoScanner database were associated with inflammatory diseases. For detailed information on SNPs related to positive results, please refer to Table 2.

**Table 1:** Summary of inflammatory cytokines causally associated with prostatitis.

Exposure	Outcome	Initial/inclusion SNPs	MR-PRESSO P-Global	F	Confounding SNPs	Palindrome sequence
IL-10RA	Prostatitis	11/9	0.805	20.45–47.99	rs10091841	rs1406714
IL-12B	Prostatitis	28/23	0.437	20.68–1162.50	rs11039216 rs117888068 rs3130510 rs3184504 rs705705	NA
CD244	Prostatitis	21/20	0.815	20.91–303.69	rs3184504	NA
uPA	Prostatitis	21/20	0.805	21.37–201.10	rs597808	NA

CD244 = Natural Killer Cell Receptor 2B4, IL-10RA = Interleukin-10 receptor A, IL-12B = Interleukin-12B, uPA = urokinase-type plasminogen activator.

**Table 2:** Summary of prostatitis causally associated with inflammatory cytokines.

Exposure	Outcome	Initial/inclusion SNPs	MR-PRESSO P-Global	F	Confounding SNPs	Palindrome sequence
Prostatitis	CCL-23	22/21	0.896	17.50–29.41	rs11642009	NA
Prostatitis	IL-5	22/21	0.956	17.50–29.41	rs11642009	NA
Prostatitis	TRANCE	22/21	0.437	17.50–29.41	rs11642009	NA

CCL23 = chemokine (C-C motif) ligand 23, IL-5 = Interleukin-5, TRANCE = TNF-related activation-induced cytokine.

### 3.2. Outcomes of Mendelian randomization

**Figure 2.**
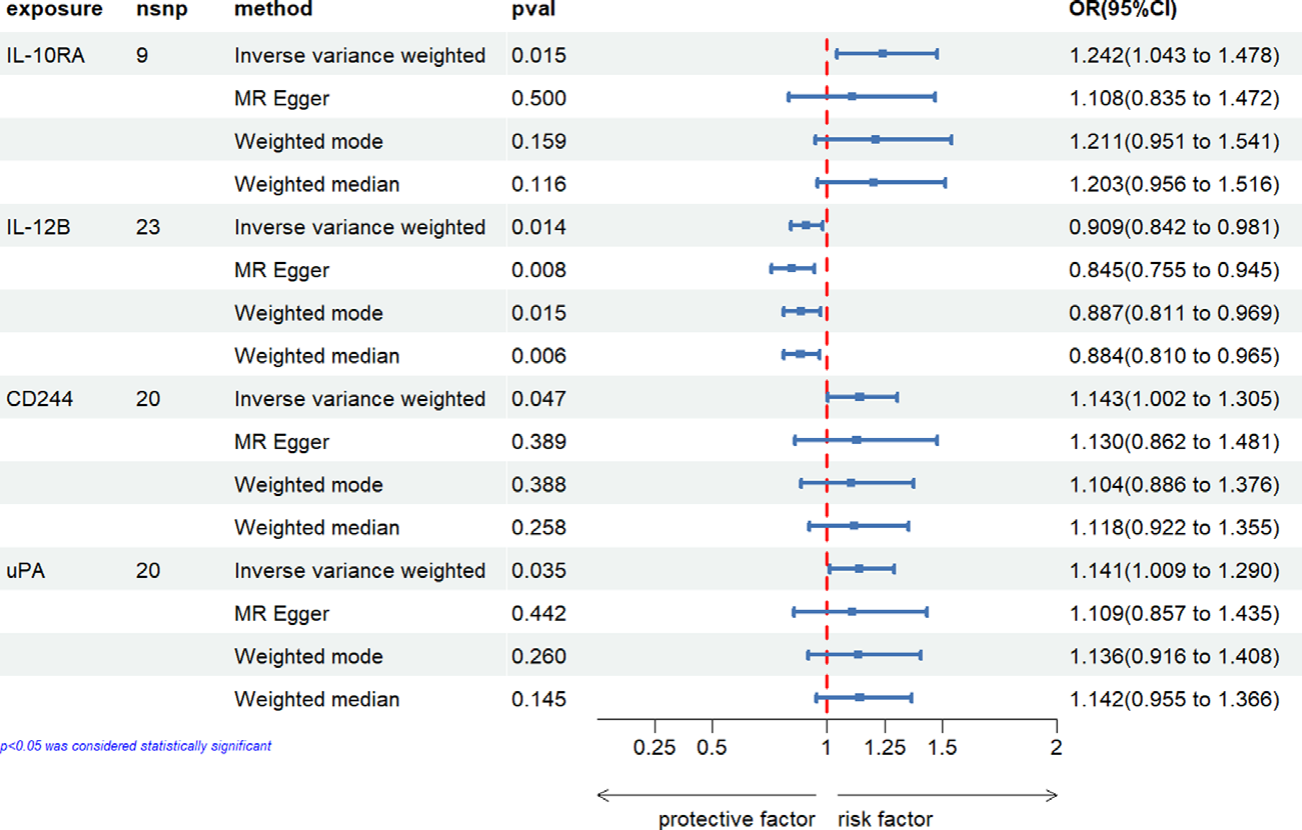
The forest plot illustrates the results of MR analysis on the impact of 91 cytokines on prostatitis. CI = confidence interval, MR analysis = Mendelian Randomization analysis, OR = odds ratio, SNP = single nucleotide polymorphism.

**Figure 3.**
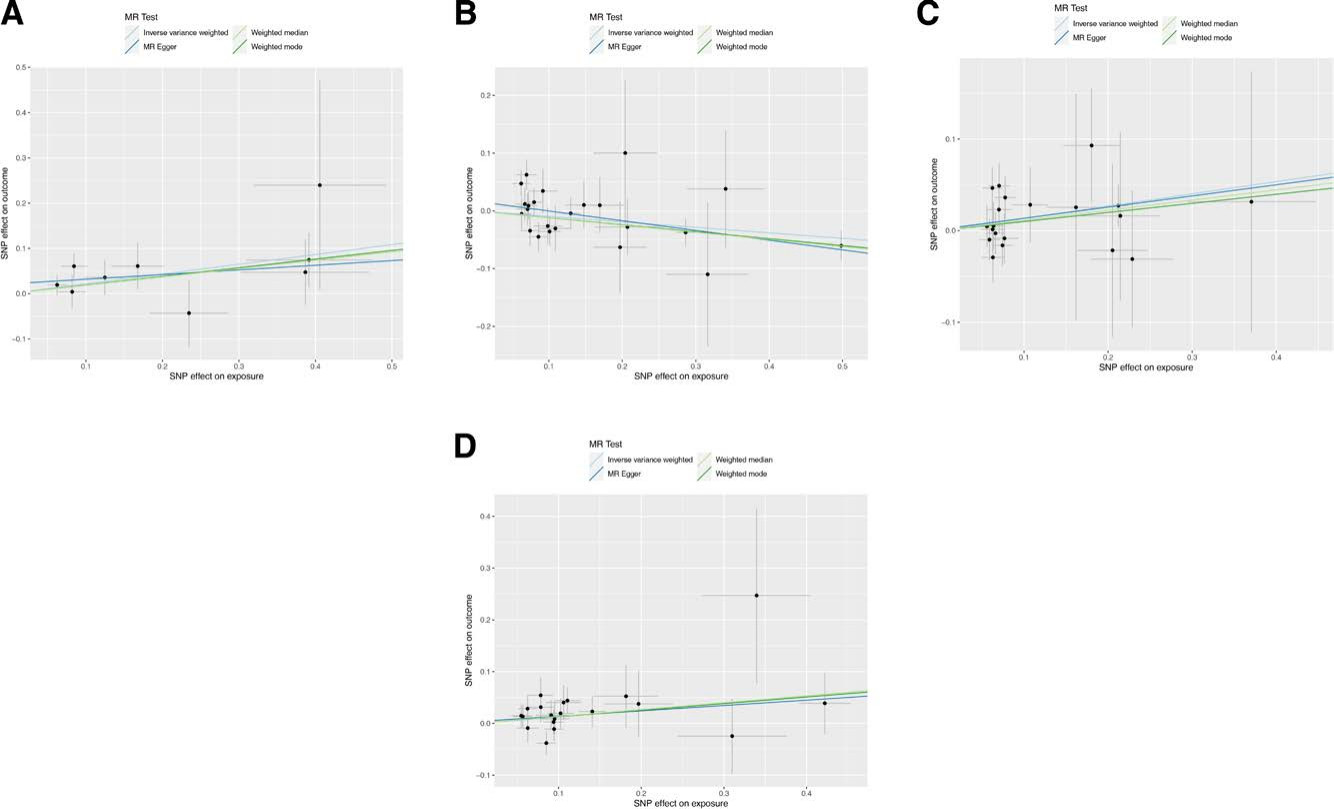
This heatmap illustrates the MR analysis outcomes concerning the influence of 91 inflammatory cytokines on prostatitis. It includes the names of all inflammatory cytokine phenotypes and the *P* values of 4 MR analyses, along with the corresponding OR values from the IVW analysis method. The outer circle represents the names of the inflammatory cytokine phenotypes, while the middle circle uses different colors to indicate the *P* value results of different MR analyses. The inner circle employs various colors to represent the OR value results from the IVW analysis. IVW = inverse variance-weighted, MR analysis = Mendelian Randomization analysis, OR = odds ratio.

**Figure 4.**
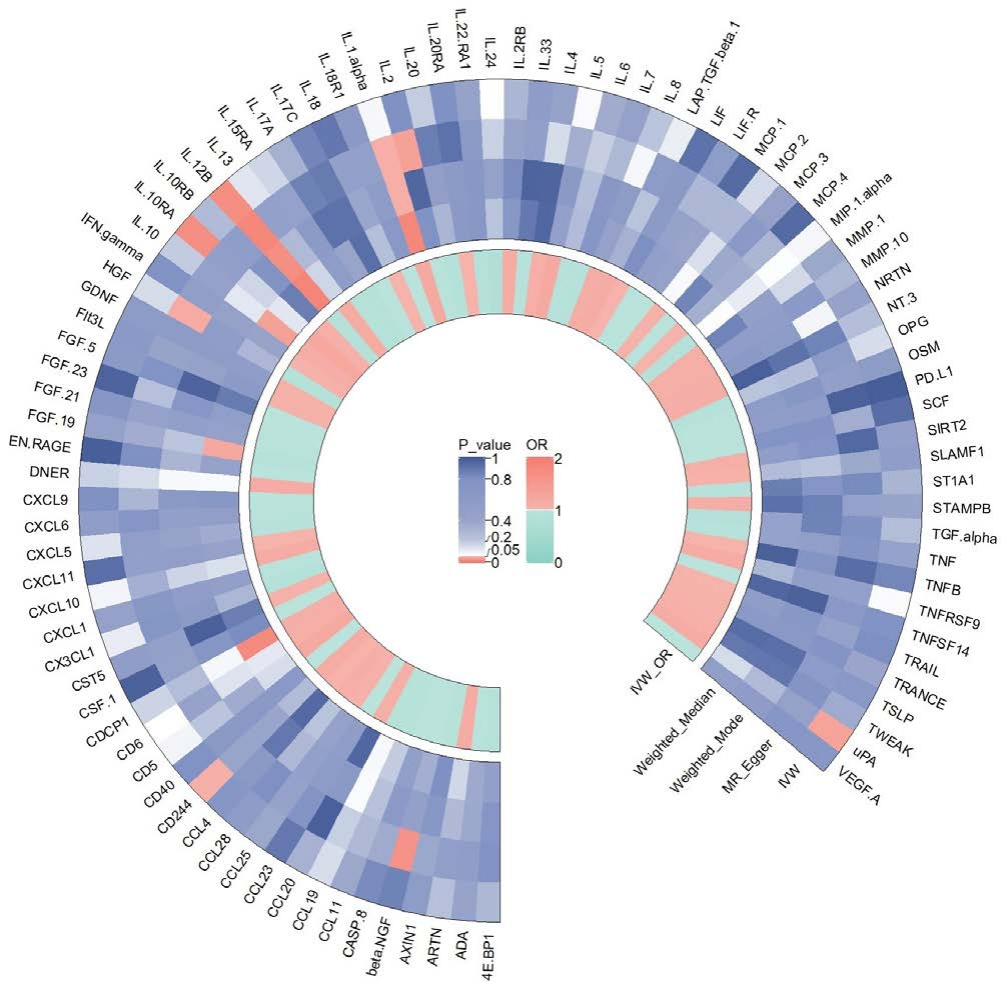
scatter plots (A) IL-10RA on prostatitis; (B) IL-12B on prostatitis; (C) CD244 on prostatitis; (D) uPA on prostatitis. CD244 = Natural Killer Cell Receptor 2B4, IL-10RA = Interleukin-10 receptor A, IL-12B = Interleukin-12B, uPA = urokinase-type plasminogen activator.

We initially investigated the impact of 91 inflammatory cytokines on prostatitis, with specific results displayed in Figure 2. The IVW method revealed causal relationships between 4 inflammatory cytokines and prostatitis. The IVW analysis results for IL-10RA (IVW_IL-10RA_: OR = 1.242, 95% CI: 1.043–1.478, *P *= .015), CD244 (IVW_CD244_: OR = 1.143, 95% CI: 1.002–1.305, *P *= .047), and uPA (IVW_uPA_: OR = 1.141, 95% CI: 1.009–1.290, *P *= .035) indicated positive associations with the risk of prostatitis. The IVW analysis result for IL-12B (IVW_IL-12B_: OR = 0.909, 95% CI: 0.842–0.981, *P *= .014) demonstrated a negative correlation with the risk of prostatitis. Notably, other results for IL-12B (MR Egger: OR = 0.845, 95% CI: 0.755–0.945, *P* = .008; weighted median: OR = 0.884, 95% CI: 0.810–0.965, *P* = .006; weighted mode: OR = 0.887, 95% CI: 0.811–0.969, *P* = .015;) also indicated a negative association with the risk of prostatitis. The scatter plots for the Mendelian randomization analysis of the aforementioned 4 inflammatory cytokines are shown in Figure 3, while all MR analysis results are presented as a heatmap in Figure 4.

**Figure 5.**
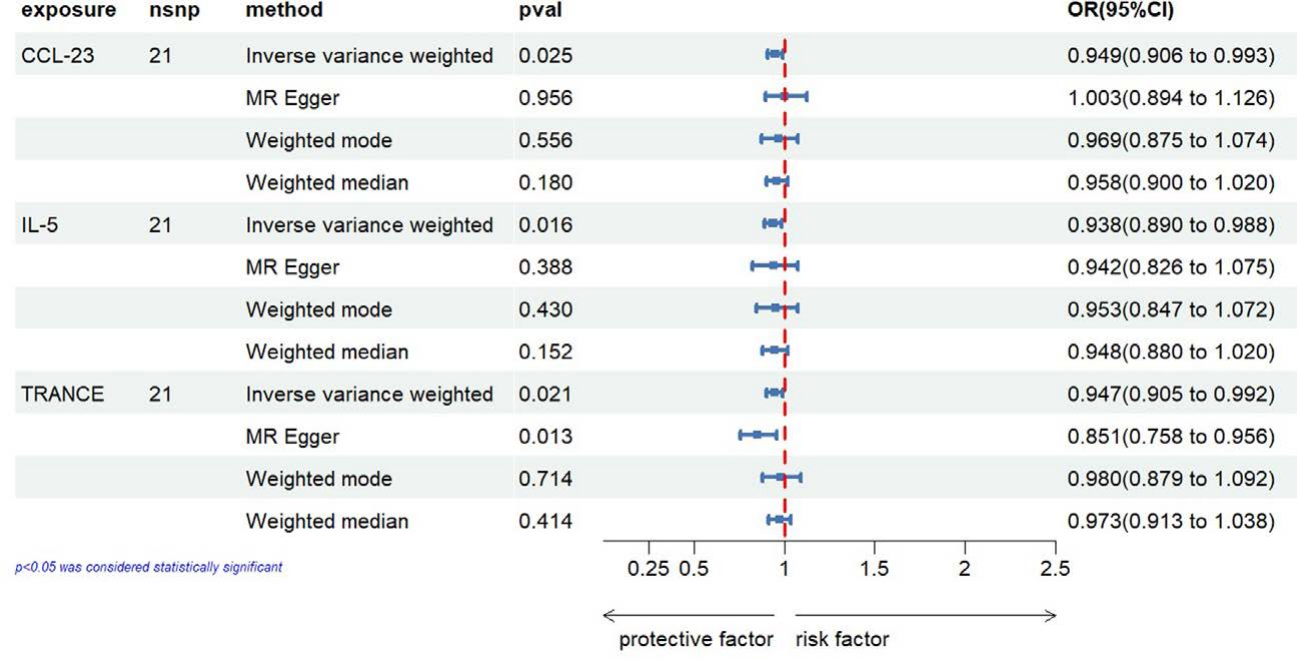
The forest plot illustrates the results of MR analysis on the impact of prostatitis on 91 cytokines. CI = confidence interval, MR analysis = Mendelian Randomization analysis, OR = odds ratio, SNP = single nucleotide polymorphism.

**Figure 6.**
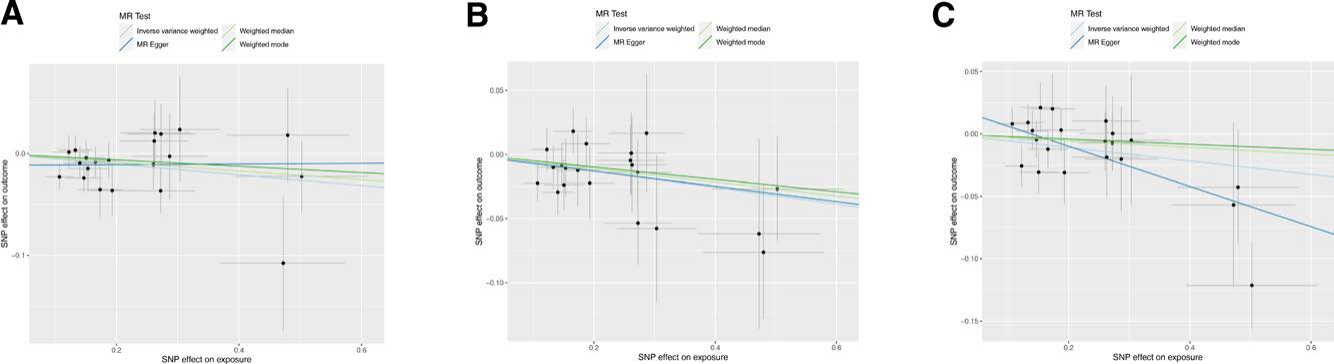
This heatmap illustrates the MR analysis outcomes concerning the influence of prostatitis on 91 inflammatory cytokines. It includes the names of all inflammatory cytokine phenotypes and the *P* values of 4 MR analyses, along with the corresponding OR values from the IVW analysis method. The outer circle represents the names of the inflammatory cytokine phenotypes, while the middle circle uses different colors to indicate the *P* value results of different MR analyses. The inner circle employs various colors to represent the OR value results from the IVW analysis. IVW = inverse variance-weighted, MR analysis = Mendelian Randomization analysis, OR = odds ratio.

**Figure 7.**
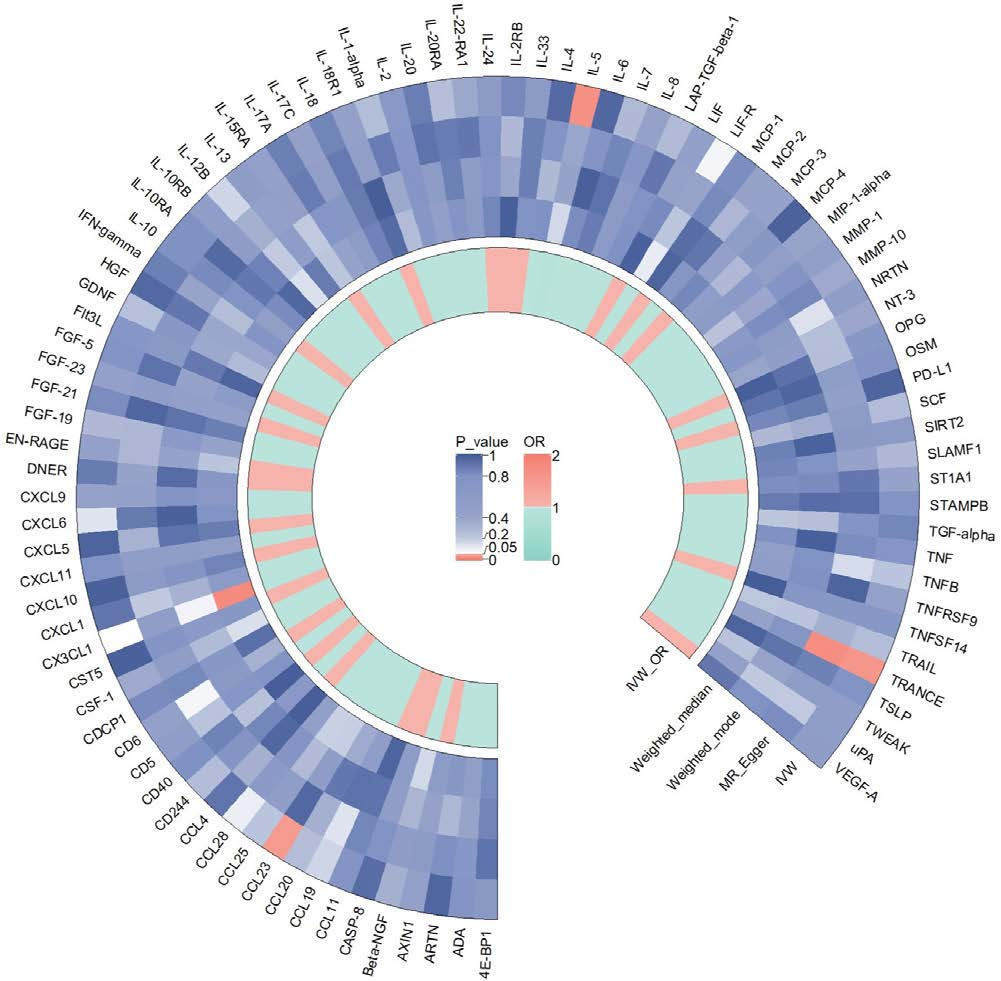
Scatter plots (A) prostatitis on CCL23; (B) prostatitis on IL-5; (C) prostatitis on TRANCE. CCL23 = chemokine (C-C motif) ligand 23, IL-5 = Interleukin-5, TRANCE = TNF-related activation-induced cytokine.

For the reverse MR results, they are displayed in Figure 5. The IVW method revealed causal relationships between prostatitis and 3 inflammatory cytokines. The IVW analysis results for CCL23 (IVW_CCL23_: OR = 0.949, 95% CI: 0.906–0.993, *P *= .025), IL-5 (IVW_IL-5_: OR = 0.938, 95% CI: 0.890–0.988, *P *= .016), and TRANCE (IVW_TRANCE_: OR = 0.947, 95% CI: 0.905–0.992, *P *= .021) indicated a decrease in their expression levels in the plasma of prostatitis patients. The scatter plots for the Mendelian randomization analysis of the aforementioned 3 inflammatory cytokines are shown in Figure 6, while all reverse MR analysis results are presented as a heatmap in Figure 7.

The specific SNP information for inflammatory factors associated with prostatitis included in the MR and reverse MR analyses can be found in the File S1, Supplemental Digital Content, https://links.lww.com/MD/O884.

### 3.3. The Result of GO and KEGG pathway analyses

**Figure 8.**
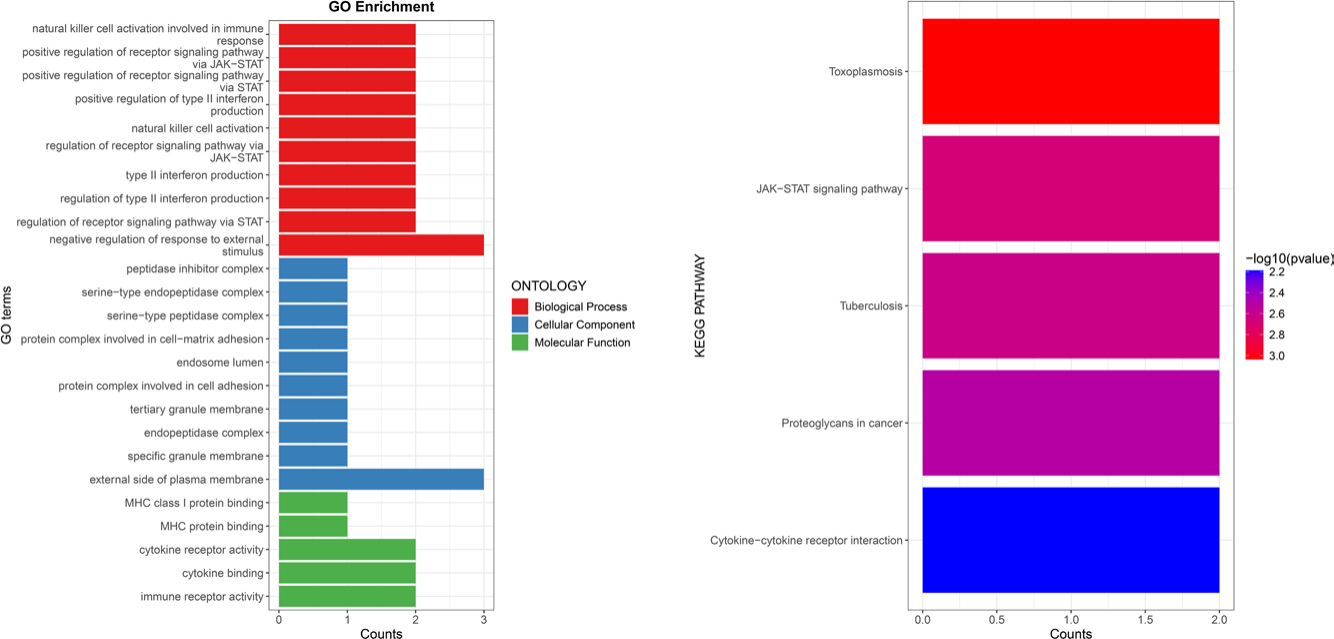
The bar plots illustrates the GO and KEGG enrichment analyses of IL-10RA, IL-12B, CD244, and uPA. CD244 = Natural Killer Cell Receptor 2B4, GO = gene ontology, IL-10RA = Interleukin-10 receptor A, IL-12B = Interleukin-12B, KEGG = kyoto encyclopedia of genes and genomes, uPA = urokinase-type plasminogen activator.

GO functional enrichment analysis revealed 25 significantly enriched GO terms, including 10 biological processes primarily involved in the regulation of the immune system, signal transduction via the JAK-STAT pathways, and the activation of natural killer (NK) cells and their roles in immune responses; 10 CC were mainly associated with the external side of the plasma membrane, granule membrane, and endosomal lumen; 5 molecular functions were primarily related to immune receptor activity and cytokine and receptor binding. KEGG pathway analysis indicated that the 4 inflammation factors positive in the MR analysis were primarily involved in the regulation of the JAK-STAT signaling pathway in immune responses and disease, as well as cytokine-cytokine receptor interactions. Figure 8 displays the results of GO terms and KEGG terms.

### 3.4. Quality assurance

**Figure 9.**
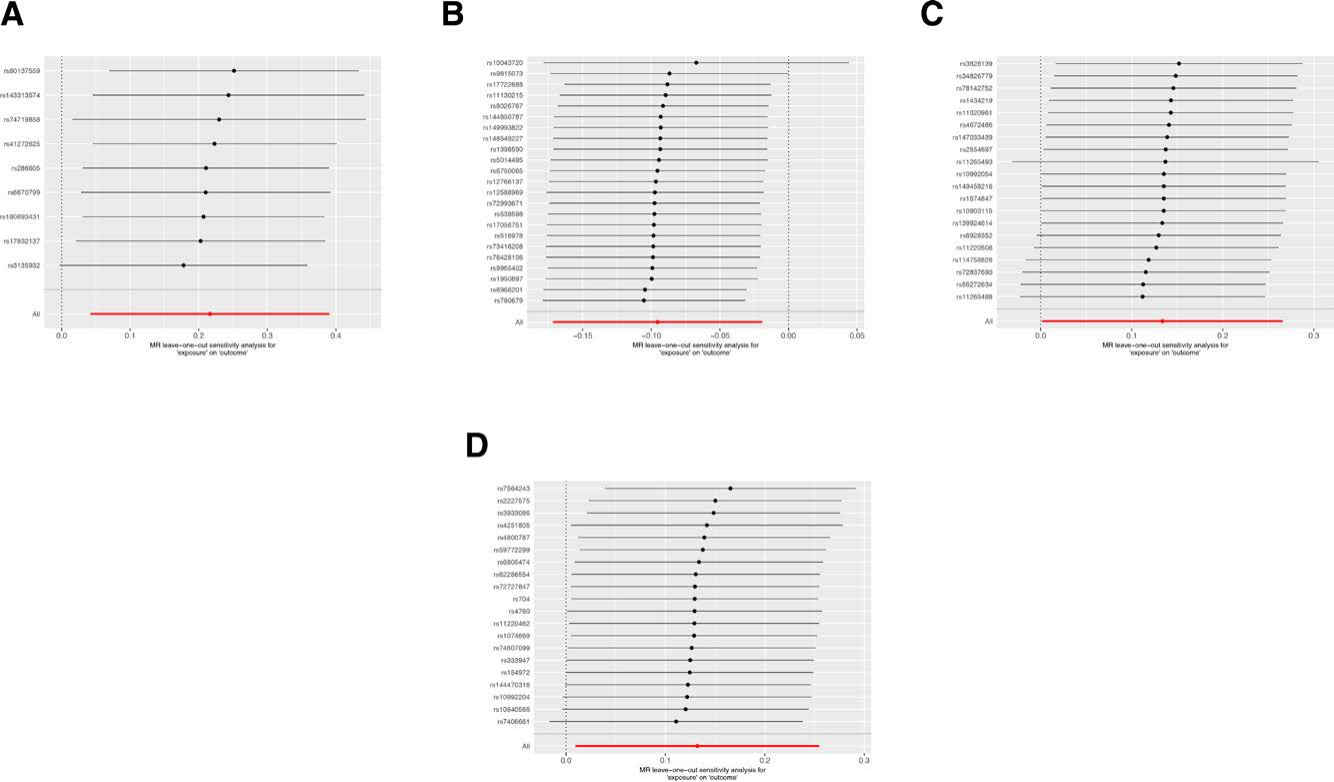
Leave-one-out analysis (A) IL-10RA on prostatitis; (B) IL-12B on prostatitis; (C) CD244 on prostatitis; (D) uPA on prostatitis. CD244 = Natural Killer Cell Receptor 2B4, IL-10RA = Interleukin-10 receptor A, IL-12B = Interleukin-12B, IL-10RA = Interleukin-10 receptor A, IL-12B = Interleukin-12B,

**Figure 10.**
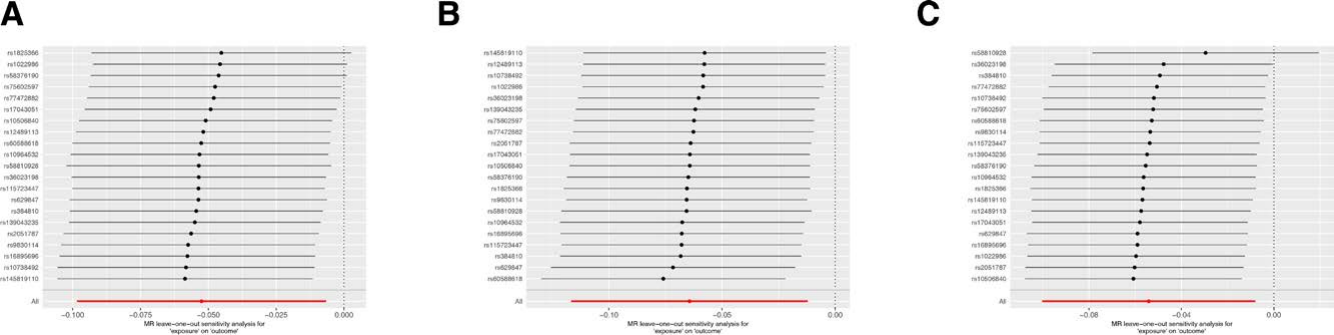
Leave-one-out analysis (A) prostatitis on CCL23; (B) prostatitis on IL-5; (C) prostatitis on TRANCE. CCL23 = chemokine (C-C motif) ligand 23, IL-5 = Interleukin-5, TRANCE = TNF-related activation-induced cytokine.

To assess the robustness of our study results, we conducted sensitivity analyses, including MR-Egger regression and Cochran’s Q test. The MR analysis results are presented in Table 3, and the reverse results are shown in Table 4. Our findings indicate no evidence of heterogeneity or pleiotropy in the study. Further validation of the consistency of results was achieved through leave-one-out analysis, where SNPs were sequentially removed and MR estimates were reassessed. Figure 9 displays the leave-one-out analysis plots for the 4 inflammatory cytokines obtained from the MR analysis, while Figure 10 shows the leave-one-out analysis plots for the 3 inflammatory cytokines obtained from the reverse MR analysis. The previously conducted MR-PRESSO analysis further confirmed this consistency. Overall, our study’s findings exhibit robustness through these analyses.

**Table 3:** Pleiotropy and heterogeneity test for inflammatory cytokines on prostatitis.

Exposure	Outcome	Pleiotropy test			Heterogeneity test Cochran’ Q			
		MR-Egger-intercept	MR-Egger_P	MR-PRESSO_P	MR Egger_Q	MR Egger_P	IVW_Q	IVW_ P
IL-10RA	Prostatitis	0.022	0.353	0.805	4.103	0.768	5.092	0.748
IL-12B	Prostatitis	0.017	0.106	0.437	20.823	0.470	23.670	0.365
CD244	Prostatitis	0.001	0.922	0.815	13.892	0.736	13.902	0.789
uPA	Prostatitis	0.003	0.808	0.805	13.776	0.744	13.837	0.793

CD244 = Natural Killer Cell Receptor 2B4, IL-10RA = Interleukin-10 receptor A, IL-12B = Interleukin-12B, uPA = urokinase-type plasminogen activator.

**Table 4:** Pleiotropy and heterogeneity test for prostatitis on inflammatory cytokines.

Exposure	Outcome	Pleiotropy test			Heterogeneity test Cochran’ Q			
		MR-Egger-intercept	MR-Egger_P	MR-PRESSO_P	MR Egger_Q	MR Egger_P	IVW_Q	IVW_ P
Prostatitis	CCL23	−0.012	0.315	0.896	12.457	0.865	13.522	0.854
Prostatitis	IL-5	−0.001	0.938	0.956	11.053	0.922	11.059	0.945
Prostatitis	TRANCE	0.022	0.063	0.437	15.826	0.669	19.731	0.475

CCL23 = chemokine (C-C motif) ligand 23, IL-5 = Interleukin-5, TRANCE = TNF-related activation-induced cytokine.

## 4. Discussion

Our study indicates that in MR analysis, 4 inflammatory cytokines are associated with the development of prostatitis. Interleukin-10 receptor A (IL-10RA), Natural Killer Cell Receptor 2B4 (CD244), and urokinase-type plasminogen activator (uPA) may contribute to the occurrence of prostatitis, while Interleukin-12B (IL-12B) appears to serve as a protective factor against it. Reverse MR analysis suggests that prostatitis can reduce the levels of 3 inflammatory cytokines: chemokine (C-C motif) ligand 23 (CCL23), IL-5, and TNF-related activation-induced cytokine (TRANCE).

In prostatitis, CP/CPPS is considered the most common and prevalent type, representing the highest proportion among prostatitis cases. Currently, there is no definitive gold standard for diagnosing CP/CPPS because bacterial cultures of prostatic secretions in CP/CPPS patients are typically negative, yet it is still believed that other pathogenic microorganisms may cause the infection.^[2]^ Common examples include chlamydia and viruses among other pathogenic microorganisms,^[2,18,19]^ all of which reside intracellularly. Due to the noninvasive nature of prostatitis examinations, it is challenging to detect intracellular pathogens. Studies suggest that after infection with chlamydia and viruses, they are difficult to completely clear and can persist in the human body for prolonged periods.^[20]^ The persistent infection of these pathogens in prostate cells indicates their immune evasion capability, closely linked to the cytokines secreted by immune cells.

The IL-10 receptor is composed of IL-10RA dimers and IL-10 receptor B (IL-10RB) dimers. IL-10RA specifically binds to IL-10, while IL-10RB, due to its low affinity, can bind not only to IL-10 but also to other cytokines in the IL-10 family.^[21,22]^ IL-10 is considered a factor in inflammation and immune suppression, and it also regulates cell growth and differentiation, making it a multifunctional cytokine. IL-10 may lead to chronic inflammation by inhibiting the proliferation of CD4+ and CD8+ T cells,^[23]^ potentially resulting in the infection of latent pathogens such as Chlamydia trachomatis and viruses within the prostate or reproductive tract.^[24,25]^ MR analysis indicates no causal relationship between IL-10, IL-10RB, and prostatitis, while IL-10RA is identified as a risk factor for prostatitis. The differences in MR analysis results may be related to their affinity in IL-10 signaling. IL-10, due to its specific binding and activation of IL-10RA, subsequently activates the JAK-STAT3 pathway, phosphorylating kinase Jak1 to exert its biological effects, inhibiting Th1 cells, and thereby suppressing the immune system’s clearance of pathogens.^[21]^ Therefore, modulating the interaction between IL-10 and the IL-10RA receptor could be a potential therapeutic approach for CP/CPPS.

uPA is a cytokine capable of activating the fibrinolysis system, promoting cell migration, and regulating inflammation. Numerous studies have elucidated that uPA promotes cell migration and the spread of prostate cancer.^[26–28]^ In this study, MR analysis demonstrated that uPA is a risk factor for prostatitis, a condition considered to be a risk factor for prostate cancer.^[29]^ Guo et al^[30]^ discovered that uPA-activated plasminogen can activate the STAT3 protein. Studies have shown that STAT3 is a key factor in IL-10 signal transduction.^[21]^ Through this factor, uPA can also exert the same immunosuppressive effect as IL-10, leading to persistent chronic infections in the prostate. The aforementioned literature provides evidence that uPA plays multiple roles in the pathogenesis of prostate diseases. Consequently, targeting uPA through interventions may present novel therapeutic strategies and mechanisms for managing CP/CPPS.

CD244 is present on the surface of NK cells, T cells, eosinophils, monocytes, dendritic cells, and myeloid-derived suppressor cells, where it binds with its ligand CD48 to exert immunological effects. MR analysis indicates that CD244 is a risk factor for prostatitis. Studies have revealed that the prostasomes secreted by prostatic epithelial cells contain a significant amount of CD48. Culturing NK cells in the granular environment of prostasomes leads to a decrease in their activity.^[31]^ NK cells with high activity levels are believed to prevent CP/CPPS.^[32]^ Therefore, CD244 may promote the development of CP/CPPS by inhibiting NK cell activity and weakening their cytolytic function. Research by Chlewicki et al^[33]^ indicates that the functionality of NK cells is determined by the level of CD244-CD48 cross-linking, with high surface density of CD244 on NK cells considered to inhibit their activity. Additionally, studies demonstrate that Epstein-Barr virus upregulates the expression of surface CD48 on infected cells to facilitate binding with CD244,^[34]^ thus increasing cross-linking levels and preventing NK cells from attacking virus-infected cells. It can be speculated that in some virus-induced prostatitis cases, CD244 may also be involved in the generation of a viral immune evasion environment, necessitating further research into its related mechanisms.

The IL-12B gene encodes the p40 subunit of IL-12. IL-12 is a heterodimeric molecule composed of p35 and p40 subunits.^[35]^ Its binding to the IL-12 receptor activates the JAK-STAT4 pathway, which further stimulates the activation of CD4+ T cells, CD8+ T cells, and NK cells.^[36]^ MR analysis indicates that IL-12B acts as an inhibitory factor for prostatitis, likely due to its role in inducing the differentiation of CD4+ T cells into Th1 and Th17 effector cells,^[37]^ as well as enhancing NK cell activity. It inhibits the development of CP/CPPS by promoting the clearance of intracellular pathogens. Moreover, the negative feedback product of the IL-12 immune response is IL-10,^[38]^ whose receptor, IL-10RA, has been identified as a potential risk factor associated with CP/CPPS. Therefore, by regulating the negative feedback product IL-10 of IL-12 and preventing its adverse effects mediated through IL-10RA, the therapeutic efficacy against CP/CPPS can be enhanced. This strategy may provide a new therapeutic direction for patients with CP/CPPS.

GO and KEGG pathway analyses reveal that genes related to IL-10RA, uPA, CD244, and IL-12B primarily regulate NK cell activity and the JAK-STAT signaling pathway. Combined with the above content, the critical roles of these genes in immune regulation are further clarified, particularly in regulating immune receptor activity, cytokine signaling, and NK cell function. Consistent with previous analyses of inflammatory factors, these findings not only support our enrichment analysis results but also provide strong evidence for understanding the role of the immune system in the pathogenesis of CP/CPPS, particularly in exploring the impact of NK cell function and the JAK-STAT signaling pathway.

Currently, no studies have investigated the mechanisms underlying the reduction in blood levels of CCL23, IL-5, and TRANCE, as identified in the results of reverse MR analysis. Nevertheless, we propose several potential mechanisms that may be associated with decreased levels of these 3 inflammatory cytokines in CP/CPPS. We hypothesize that in CP/CPPS, pathogens utilize the immune suppression induced by the interaction between IL-10 and IL-10RA, reducing the immune response of Th2 cells, thereby causing a reduction in plasma levels of CCL23. Current research on CCL23 in hepatocellular carcinoma cells has confirmed that low levels of Th2 cell activity lead to decreased expression of CCL23, resulting in impaired cell lytic function of Th1 cells.^[39]^ The study conducted by Goetzl et al^[40]^ demonstrates that the decreased levels of IL-5 are also a result of reduced Th2 cell immune responses. Additionally, studies on Hepatitis B virus indicate that low expression levels of IL-5 in plasma may reflect high levels of HBV replication.^[41]^ This study suggests that in virus-related CP/CPPS, low levels of IL-5 may also result from high levels of virus replication. The expression levels of TRANCE are positively correlated with Th1 cell immune responses.^[42]^ Therefore, in the progression of CP/CPPS, the decreased expression levels of TRANCE may be due to the suppression of the Th1 immune response by the interaction between IL-10 and IL-10RA. In summary, further research is needed to validate our findings and explore the potential mechanisms involved in the aforementioned possible outcomes.

This study elucidated the relationship between prostatitis and 91 inflammatory cytokines through Mendelian randomization studies. The advantage lies in the ability of MR studies to weaken reverse causation and bias caused by confounding. Additionally, the study population was limited to individuals of European ancestry, reducing bias resulting from ethnicity. However, the study also has limitations. Firstly, the data we selected were from two GWAS, where detailed records of prostatitis-related symptoms in patients were lacking, preventing deeper subgroup analysis. Secondly, due to the homogeneity of the population, the global applicability of the research findings remains to be verified. Overall, with the support of large sample size MR analysis, we believe that the estimated effects will approximate reality. However, more research is needed to validate and apply them in clinical practice.

## 5. Conclusions

In summary, this study provides new evidence for the mutual relationship between prostatitis and circulating inflammatory cytokines. It emphasizes the bidirectional causal relationships that specific circulating inflammatory cytokines may have with prostatitis, as well as the potential of acupuncture in modulating immune responses for the treatment of prostatitis. These findings provide deeper understanding into the pathogenesis of prostatitis, offering valuable insights for advancing targeted therapies in the future. However, further research is needed to validate these findings and explore potential biological mechanisms.

## Acknowledgments

The authors extend sincere gratitude to the investigators who generously provided the GWAS summary statistics utilized in this study

## Author contributions

**Conceptualization:** Jiaqi Ma, Lilei Xu.

**Data curation:** Jiaqi Ma, Lilei Xu, Chuanlong Zhou.

**Formal analysis:** Jiaqi Ma, Lilei Xu.

**Funding acquisition:** Chuanlong Zhou.

**Investigation:** Jiaqi Ma.

**Methodology:** Jiaqi Ma, Zhe Shen.

**Project administration:** Zhe Shen, Kean Zhu.

**Resources:** Jiaqi Ma, Zhe Shen, Kean Zhu.

**Software:** Jiaqi Ma, Lilei Xu, Kean Zhu.

**Supervision:** Zhe Shen, Kean Zhu, Xianming Lin.

**Validation:** Jiaqi Ma.

**Visualization:** Jiaqi Ma.

**Writing – original draft:** Jiaqi Ma, Lilei Xu, Chuanlong Zhou.

**Writing – review & editing:** Jiaqi Ma, Lilei Xu, Chuanlong Zhou, Xianming Lin.

## Correction

This article was originally published with an incorrect funding information has now been corrected online from “*This article is funded by the Special Funds for Co-construction Projects between the Science and Technology Department of the State Administration of Traditional Chinese Medicine and the Zhejiang Provincial Administration of Traditional Chinese Medicine. (Grant Number: Traditional Chinese Medicine Science and Technology Letter [2023] no. 139).*”to “*This article is funded by the Special Funds for Co-construction Projects between the Science and Technology Department of the State Administration of Traditional Chinese Medicine and the Zhejiang Provincial Administration of Traditional Chinese Medicine. (Grant Number: Traditional Chinese Medicine Science and Technology Letter [2023] no. 139: GZY-ZJ-KJ-24054).*”

## Supplementary Material



## References

[1] Pendegast HJ. Chronic prostatitis and chronic pelvic pain syndrome in men. In: StatPearls. U.S. National Library of Medicine; 2024. https://pubmed.ncbi.nlm.nih.gov/38261706/. Accessed February 1, 2024.

[2] Khan FU Ihsan AU Khan HU. Comprehensive overview of prostatitis. Biomed Pharmacother. 2017;94:1064–76. 28813783 10.1016/j.biopha.2017.08.016

[3] Pontari MA. Etiologic theories of chronic prostatitis/chronic pelvic pain syndrome. Curr Urol Rep. 2007;8:307–12. 18519015 10.1007/s11934-007-0077-6

[4] Motrich RD Salazar FC Breser ML. Implications of prostate inflammation on male fertility. Andrologia. 2018;50:e13093. 30569650 10.1111/and.13093

[5] Graziani A Grande G Martin M. Chronic prostatitis/chronic pain pelvic syndrome and male infertility. Life (Basel). 2023;13:1700. 37629557 10.3390/life13081700 PMC10455764

[6] Condorelli RA Russo GI Calogero AE Morgia G La Vignera S. Chronic prostatitis and its detrimental impact on sperm parameters: a systematic review and meta-analysis. J Endocrinol Invest. 2017;40:1209–18. 28488229 10.1007/s40618-017-0684-0

[7] Berg E Houska P Nesheim N. Chronic prostatitis/chronic pelvic pain syndrome leads to impaired semen parameters, increased sperm DNA fragmentation and unfavorable changes of sperm protamine mRNA ratio. Int J Mol Sci. 2021;22:7854. 34360620 10.3390/ijms22157854 PMC8346101

[8] Havrylyuk A Chopyak V Boyko Y Kril I Kurpisz M. Cytokines in the blood and semen of infertile patients. Cent Eur J Immunol. 2015;40:337–44. 26648778 10.5114/ceji.2015.54596 PMC4655384

[9] Yin L Tang Y Pan A Yang L Zhu X Liu Y. The application of IL-10 and TNF-α in expressed prostatic secretions and prostatic exosomal protein in urine in the diagnosis of patients with chronic prostatitis. Medicine (Baltimore). 2019;98:e16848. 31415412 10.1097/MD.0000000000016848 PMC6831336

[10] He L Wang Y Long Z Jiang C. Clinical significance of IL-2, IL-10, and TNF-α in prostatic secretion of patients with chronic prostatitis. Urology. 2010;75:654–7. 19963254 10.1016/j.urology.2009.09.061

[11] Nadler RB Koch AE Calhoun EA. IL-1β and TNF-α in prostatic secretions are indicators in the evaluation of men with chronic prostatitis. J Urol. 2000;164:214–8. 10840462

[12] Davey Smith G Hemani G. Mendelian randomization: Genetic anchors for causal inference in epidemiological studies. Hum Mol Genet. 2014;23:R89–98. 25064373 10.1093/hmg/ddu328 PMC4170722

[13] Davies NM Holmes MV Davey Smith G. Reading Mendelian randomisation studies: a guide, glossary, and checklist for Clinicians. BMJ. 2018;362:k601. 30002074 10.1136/bmj.k601 PMC6041728

[14] Zhang Y Peng R Chen Z. Evidence for a causal effect of major depressive disorder, anxiety on prostatitis risk: a univariate and multivariate Mendelian randomization study. Prostate. 2023;83:1387–92. 37504798 10.1002/pros.24601

[15] Zhao JH Stacey D Eriksson N.; Estonian Biobank Research Team. Genetics of circulating inflammatory proteins identifies drivers of immune-mediated disease risk and therapeutic targets. Nat Immunol. 2023;24:1540–51. 37563310 10.1038/s41590-023-01588-w PMC10457199

[16] Han Z Tian R Ren P. Parkinson’s disease and alzheimer’s disease: a mendelian randomization study. BMC Med Genet. 2018;19(Suppl 1):215. 30598082 10.1186/s12881-018-0721-7 PMC6311900

[17] Bowden J Davey Smith G Burgess S. Mendelian randomization with invalid instruments: effect estimation and bias detection through Egger regression. Int J Epidemiol. 2015;44:512–25. 26050253 10.1093/ije/dyv080 PMC4469799

[18] Dan M Siegel YI Korczak D Lindner A Samra Z. Isolation of chlamydia trachomatis from prostatic tissue of patients undergoing transurethral prostatectomy. Infection. 1991;19:162–3. 1889870 10.1007/BF01643241

[19] Weidner W Diemer T Huwe P Rainer H Ludwig M. The role of chlamydia trachomatis in prostatitis. Int J Antimicrob Agents. 2002;19:466–70. 12135834 10.1016/s0924-8579(02)00094-8

[20] Hodel F Naret O Bonnet C. The combined impact of persistent infections and human genetic variation on C-reactive protein levels. BMC Med. 2022;20:416. 36320076 10.1186/s12916-022-02607-7 PMC9623937

[21] Ouyang W Rutz S Crellin NK Valdez PA Hymowitz SG. Regulation and functions of the Il-10 family of cytokines in inflammation and disease. Annu Rev Immunol. 2011;29:71–109. 21166540 10.1146/annurev-immunol-031210-101312

[22] Pestka S Krause CD Sarkar D Walter MR Shi Y Fisher PB. Interleukin-10 and related cytokines and receptors. Annu Rev Immunol. 2004;22:929–79. 15032600 10.1146/annurev.immunol.22.012703.104622

[23] Ejrnaes M Filippi CM Martinic MM. Resolution of a chronic viral infection after interleukin-10 receptor blockade. J Exp Med. 2006;203:2461–72. 17030951 10.1084/jem.20061462 PMC2118120

[24] Mackern-Oberti JP Motrich RD Breser ML Sánchez LR Cuffini C Rivero VE. Chlamydia trachomatis infection of the male genital tract: an update. J Reprod Immunol. 2013;100:37–53. 23870458 10.1016/j.jri.2013.05.002

[25] Ku J-H Paick J-S Kim S-W. Chronic prostatitis in Korea: a nationwide postal survey of practicing urologists in 2004. Asian J Androl. 2005;7:427–32. 16281092 10.1111/j.1745-7262.2005.00060.x

[26] Nassir AM Kamel HFM. Explication of the roles of Prostate Health Index (PHI) and Urokinase plasminogen activator (UPA) as Diagnostic and predictor tools for prostate cancer in equivocal PSA range of 4–10 ng/ML. Saudi J Biol Sci. 2020;27:1975–84. 32714021 10.1016/j.sjbs.2020.04.004 PMC7376136

[27] Böhm L Serafin A Akudugu J Fernandez P van der Merwe A Aziz NA. UPA/pai-1 ratios distinguish benign prostatic hyperplasia and prostate cancer. J Cancer Res Clin Oncol. 2013;139:1221–8. 23595126 10.1007/s00432-013-1428-y PMC11824374

[28] Randle DD Clarke S Henderson V Odero-Marah VA. Snail mediates invasion through UPA/Upar and the MAPK signaling pathway in prostate cancer cells. Oncol Lett. 2013;6:1767–73. 26889270 10.3892/ol.2013.1635 PMC4738196

[29] Markowski MC Bowen C Gelmann EP. Inflammatory cytokines induce phosphorylation and ubiquitination of prostate suppressor protein nkx3.1. Cancer Res. 2008;68:6896–901. 18757402 10.1158/0008-5472.CAN-08-0578 PMC2586101

[30] Guo Y Li J Hagström E Ny T. Beneficial and detrimental effects of plasmin(ogen) during infection and sepsis in mice. PLoS One. 2011;6:e24774. 21931850 10.1371/journal.pone.0024774 PMC3171470

[31] Tarazona R Delgado E Guarnizo MC. Human prostasomes express CD48 and interfere with NK cell function. Immunobiology. 2011;216:41–6. 20382443 10.1016/j.imbio.2010.03.002

[32] Wang H Zhang J Ma D Zhao Z. The role of acupuncture and its related mechanism in treating chronic prostatitis/chronic pelvic pain syndrome. Int J Gen Med. 2023;16:4039–50. 37700742 10.2147/IJGM.S417066 PMC10493142

[33] Chlewicki LK Velikovsky CA Balakrishnan V Mariuzza RA Kumar V. Molecular basis of the dual functions of 2B4 (CD244). J Immunol. 2008;180:8159–67. 18523281 10.4049/jimmunol.180.12.8159

[34] Xie C Wang S Zhang H. LNC-AIFM2-1 promotes HBV immune escape by acting as a cerna for mir-330-3p to regulate CD244 expression. Front Immunol. 2023;14:1121795. 36845111 10.3389/fimmu.2023.1121795 PMC9946971

[35] Colombo MP Trinchieri G. Interleukin-12 in anti-tumor immunity and immunotherapy. Cytokine Growth Factor Rev. 2002;13:155–68. 11900991 10.1016/s1359-6101(01)00032-6

[36] Del Vecchio M Bajetta E Canova S. Interleukin-12: biological properties and clinical application. Clin Cancer Res. 2007;13:4677–85. 17699845 10.1158/1078-0432.CCR-07-0776

[37] Penna G Fibbi B Maggi M Adorini L. Prostate autoimmunity: from experimental models to clinical counterparts. Expert Rev Clin Immunol. 2009;5:577–86. 20477643 10.1586/eci.09.37

[38] Meyaard L Hovenkamp E Otto SA Miedema F. IL-12-induced IL-10 production by human T cells as a negative feedback for IL-12-induced immune responses. J Immunol. 1996;156:2776–82. 8609396

[39] Karan D. CCL23 in balancing the act of endoplasmic reticulum stress and antitumor immunity in hepatocellular carcinoma. Front Oncol. 2021;11:727583. 34671553 10.3389/fonc.2021.727583 PMC8522494

[40] Goetzl EJ. Th2 cells in rapid immune responses and protective avoidance reactions. FASEB J. 2024;38:e23485. 38372961 10.1096/fj.202302584RR

[41] Dimitriadis K Katelani S Pappa M Fragkoulis GE Androutsakos T. The role of interleukins in HBV infection: a narrative review. J Pers Med. 2023;13:1675. 38138902 10.3390/jpm13121675 PMC10744424

[42] Weitzmann MN. Bone and the immune system. Toxicol Pathol. 2017;45:911–24. 29046115 10.1177/0192623317735316 PMC5749254

